# Randomized Controlled Trial Targeting Obesity-Related Behaviors: Better Together Healthy Caswell County

**DOI:** 10.5888/pcd10.120296

**Published:** 2013-06-13

**Authors:** Jamie Zoellner, Jennie L. Hill, Karissa Grier, Clarice Chau, Donna Kopec, Bryan Price, Carolyn Dunn

**Affiliations:** Author Affiliations: Jennie L. Hill, Karissa Grier, Clarice Chau, Virginia Tech, Blacksburg, Virginia; Donna Kopec, Caswell County Senior Center, Yanceyville, North Carolina; Bryan Price, Danville City Parks and Recreation, Danville, Virginia; Carolyn Dunn, 4H Youth Development and Family and Consumer Science, North Carolina State University, Raleigh, North Carolina.

## Abstract

**Introduction:**

Collaborative and multilevel interventions to effectively address obesity-related behaviors among rural communities with health disparities can be challenging, and traditional research approaches may be unsuitable. The primary objective of our 15-week randomized controlled pilot study, which was guided by community-based participatory research (CBPR) principles, was to determine the effectiveness of providing twice-weekly access to group fitness classes, with and without weekly nutrition and physical activity education sessions, in Caswell County, North Carolina, a rural region devoid of medical and physical activity resources.

**Methods:**

Participants were randomly divided into 2 groups: group 1 was offered fitness sessions and education in healthful eating and physical activity; group 2 was offered fitness sessions only. Outcome measures were assessed at baseline and immediately after the intervention. Standardized assessment procedures, validated measures, and tests for analysis of variance were used.

**Results:**

Of 91 enrolled participants, most were African American (62%) or female (91%). Groups were not significantly different at baseline. Group 1 experienced significantly greater improvements in body mass index (*F* = 15.0, *P* < .001) and waist circumference (*F* = 7.0, *P* = .01), compared with group 2. Both groups significantly increased weekly minutes of moderate physical activity (*F* = 9.4, *P* < .003). Participants in group 1 also had significantly greater weight loss with higher attendance at the education (*F* = 14.7, *P* < .001) and fitness sessions (*F* = 18.5, *P* < .001).

**Conclusion:**

This study offers effective programmatic strategies that can reduce weight and increase physical activity and demonstrates feasibility for a larger scale CBPR obesity trial targeting underserved residents affected by health disparities. This study also signifies successful collaboration among community and academic partners engaged in a CBPR coalition.

## Introduction

Obesity is a widely recognized public health concern in the United States (1). Various individual, social, community, and environmental factors contribute to obesity-related behaviors (2). For rural areas with few resources, such as the Dan River Region in south-central Virginia and north-central North Carolina, providing collaborative and multilevel interventions to effectively address obesity-related behaviors is challenging. The Dan River Region includes Pittsylvania and Henry counties in Virginia and Caswell County in North Carolina. A rural area with health disparities, the Dan River Region is classified as a medically underserved area (3–7).

Despite the need for health evaluation data in vulnerable regions, using traditional research approaches can be difficult because of geographic location and lack of 1) community trust, 2) local health professionals and services, and 3) local qualified researchers to oversee research activities. However, the community-based participatory research (CBPR) approach can be used to overcome these obstacles. The CBPR approach is designed to build equitable community-academic partnerships, encourage community participation in all aspects of the research process, and promote program sustainability (8–10).

The intervention reported here was planned and implemented in the context of a CBPR coalition, the Dan River Partnership for a Healthy Community (DRPHC). The DRPHC’s mission is to foster community partnerships to combat obesity in the Dan River Region through healthy lifestyle initiatives. As described elsewhere, community stakeholders developed 6 obesity causal models (11). This 15-week randomized controlled study is the first pilot intervention from the physical activity priority area. The primary aim is to determine the effectiveness of providing twice-weekly access to group fitness classes, with and without weekly nutrition and physical activity education sessions. Weight and physical activity are the primary outcomes of interest; secondary outcomes are waist circumference, blood pressure, dietary behaviors, and psychosocial variables. A secondary aim is to explore relationships among attendance levels at fitness and education sessions and the anthropometric and biologic outcomes.

## Methods

The study was conducted in Caswell County, North Carolina, which is classified as an 8 on the 9-point Rural-Urban Continuum Codes (1 = urban, 9 = completely rural) (12). The median household income of $34,747 is below average for the state ($39,061) and the nation ($41,994) (13). The county is approximately 34% African American and 63% white. Caswell County has fewer than 2 recreation and fitness facilities and high rates of obesity and diabetes compared with the rest of North Carolina (14).

After a physical activity program was selected as the intervention (11), a DRPHC physical activity subcommittee was formed. Through regular committee meetings, community partners provided feedback on the design of the study, the selection of the education curriculum, logistics of providing group fitness and education classes, processes for randomization, and data assessment procedures including the selection and review of psychosocial measuring instruments. The committee held a 90-minute listening session with a convenience sample of 12 Caswell County residents (11 female, 1 male; 8 African American, 4 white). Semistructured questions were used to ask residents about their preferences for session days and times, topics of interest, types of fitness offerings, recruitment methods, barriers to program participation, participant accountability, and data collection procedures.

### Recruitment procedures and eligibility

Participants were recruited through an advertisement in the local paper, flyers posted around town, and word of mouth. Interested community members called the local health department and were screened. Eligibility criteria were being aged 18 or older, speaking English, and having no self-reported exercise contraindications.

### Study design and intervention

Participants in this 15-week randomized controlled pilot study were randomly assigned to 1 of 2 groups. All participants received access to 2 weekly group fitness classes, offered free of charge at the Caswell County Parks and Recreation Building. Zumba classes were offered 1 night a week, and the other evening was a “potluck” night designed to increase participants’ exposure to other classes (eg, aerobics, kickboxing, line dancing). Participants in the intervention group (group 1) were enrolled in Eat Smart, Move More, Weigh Less (ESMMWL) and received weekly 1-hour classes on healthy eating and physical activity (15,16). The ESMMWL curriculum was established on evidence-based weight loss principles and strategies. The curriculum is guided by the theory of planned behavior, which empowers and motivates participants to live mindfully as they make choices about eating and physical activity (17). Each lesson includes discussion related to 1) a behavior (eg, controlling or decreasing portion sizes; eating more meals at home; increasing physical activity) and its importance to the participant’s weight goal; 2) how family and friends can support the behavior change; and 3) strategies for adopting the behavior (eg, interpreting food labels, keeping a food and physical activity record). The program has been field tested and disseminated through the North Carolina extension system; however, this study is the first known to document effects in a randomized controlled study (15,16). All phases of this research were approved by Virginia Tech’s institutional review board, and participants provided written informed consent forms.

Attendance was tracked at all sessions. Among the 50% of participants who provided e-mail addresses, 10 reminder e-mails were sent via a listserve. Participants who were absent for 2 or more consecutive weeks received approximately 1 weekly reminder or encouragement telephone call or a personalized e-mail. Raffle prizes (eg, measuring utensils, exercise DVDs) to reward attendance were provided at regular intervals in both the group fitness and education session.

Education sessions were led by 1 Caswell County Health Department employee who had completed certification for the ESMMWL curriculum. The fitness sessions were delivered by local experienced instructors, including 1 who taught Zumba and 3 others who led the potluck night.

### Outcome measures and randomization

All outcome measures were assessed at baseline and immediately after the intervention. A data collection manual of procedures was developed to standardize all assessments. Data were collected in person, in a private setting, and questionnaires were read aloud by trained and certified research staff who were blinded to the participants’ group assignment. To promote transparency in the randomization procedures, an equal number of cards marked “group 1” or “group 2” were concealed in an envelope, and participants drew their own random assignment at the end of baseline enrollment. Participants were provided a $10 gift card for completing each assessment time point.

### Anthropometric variables and blood pressure

Height, weight, waist circumference, and blood pressure were measured, respectively, with a portable stadiometer, Tanita body fat analyzer model TBF-310GS (Tanita, Arlington Heights, Illinois), nonstretchable flexible measuring tape, and an OMRON HEM-907XL (OMRON Group, Lake Forest, Illinois) automatic inflation sphygmomanometer. Participants received a personal assessment that compared their values with healthy ranges.

### Self-reported variables

The valid and reliable Godin measure and the National Cancer Institute’s Five-Factor Screener were used to assess self-reported physical activity and nutrition behaviors (18–20). Health-related quality of life was assessed with the Centers for Disease Control and Prevention’s (CDC’s) Healthy Days core module (http://www.cdc.gov/hrqol/hrqol14_measure.htm). The validated Newest Vital Sign was used to assess health literacy (21). Validated psychosocial measuring instruments (22,23) were used, including instruments to measure self-efficacy and social support for both physical activity and nutrition. Cronbach’s alphas calculated on the baseline data indicated strong internal consistency for each psychosocial scale (ɑ ≥ .90). The follow-up data collection concluded with 7 open-ended questions to explore participants’ opinions of the project and to inform future programming.

### Data analyses

Descriptive statistics were used to summarize variables. Tests for analysis of variance (ANOVA) and generalized linear models were used to examine effects. Two analytical approaches were used: an intent-to-treat analysis that uses the last observation carried forward method (eg, for noncompleters, baseline value is substituted for postintervention value [assumes a zero change]), and complete cases-only analysis (24). Findings did not vary by approach; therefore, intent-to-treat results are presented. Analyses were performed using SPSS 20.0 software (IBM, Chicago, Illinois). A critical value of ɑ = .05 was adopted for significance testing. For the open-ended questions, comments were first coded as specific to group fitness, to education, or nonspecific. Comments were then further coded as positive or negative and subsequently examined for emerging themes.

## Results

**Figure 1:**
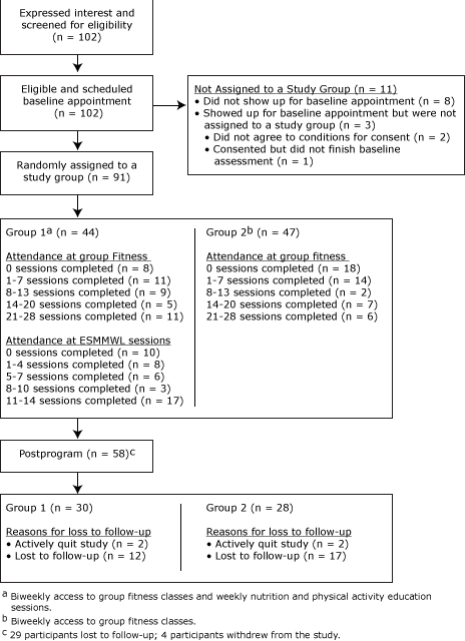
Recruitment, screening, and participation in Better Together Healthy Caswell County, North Carolina, 2011.

Of the 102 people who called to inquire about the study, all were screened, met the screening inclusion criteria, and were scheduled for an enrollment appointment (Figure 1). At enrollment, 2 participants with blood pressure higher than 180/110 mm Hg were referred for immediate medical attention; both received medical release and were enrolled. In total, 91 participants completed enrollment and were randomly assigned to a group in the trial (44 in group 1 and 47 in group 2). Attendance at group fitness sessions averaged 10.3 of 28 sessions (standard deviation [SD], 9.6) for group 1 and 7.6 of 28 (SD, 8.7) for group 2 (*F* = 1.85; *P* = 0.18) (Figure 1). Attendance at education sessions for group 1 averaged 6.6 (SD, 5.4) of 14 sessions. Postintervention data was available on 58 participants (64%).

Most participants were women (91%) and African American (62%) (Table 1). Education and income levels indicate a broad representation of socioeconomic status. Most participants were obese (31%) or morbidly obese (49%). Approximately 40% of the participants had a high likelihood of limited health literacy. No significant group differences were noted at baseline. Furthermore, demographics were not significantly different among postintervention completers and noncompleters.

**Table 1:** Demographic Characteristics of Participants at Baseline (N = 91), Better Together Healthy Caswell County (North Carolina), 2011

Characteristic	Group 1^a^ (n = 44)	Group 2^b^ (n = 47)	*P* Value^c^
**Sex**			
Female	38	45	.11
Male	6	2	
**Race**			
African American	25	31	.37
White	19	16	
**Education level**			
High school graduate or less	16	18	.98
Some college	15	16	
College degree	13	13	
**Annual income, $**			
<19,999	10	14	.90
20,000-49,999	22	21	
≥50,000	11	11	
Did not answer	1	1	
**Body mass index group**			
Normal (18.5–24.9 kg/m^2^)	1	0	.65
Overweight (25–29.9 kg/m^2^)	8	9	
Obese (30–34.9 kg/m^2^)	15	13	
Morbidly obese (≥35 kg/m^2^)	20	25	
**Health literacy^d^**			
High likelihood of limited health literacy	5	11	.22
Possibility of limited health literacy	11	7	
Adequate health literacy	28	29	

a Twice-weekly access to group fitness classes plus weekly nutrition and physical activity education sessions.

b Twice-weekly access to group fitness classes.

c χ^2^ tests.

d Assessed using the Newest Vital Sign (21): 0–1 correct answer, high likelihood of limited literacy; 2–3 correct answers, possibility of limited literacy; and 4–6 correct answers, adequate literacy skills.

Body mass index and waist circumference improved significantly between baseline and follow-up (Table 2). Group 1 participants achieved significantly greater improvements in BMI and waist circumference than did those in group 2. We found no significant effects on blood pressure. For self-reported physical activity, moderate activity increased from baseline to follow-up, yet we found no significant differences between groups on the physical activity measures. For self-reported dietary intake, with the exception of a decrease in amount of sugar used, we noted no changes in dietary variables. Significant time effects were seen for self-efficacy for physical activity (decrease) and friend support for healthy eating (increase); however, there were no significant effects for other psychosocial variables.

**Table 2:** Overall and Between Group Effects for Anthropometrics, Blood Pressure, Physical Activity, Dietary Intake, and Psychosocial Constructs (N = 91), Better Together Healthy Caswell County (North Carolina), 2011

Variable	Group 1^a^ (n = 44)		Group 2^b^ (n = 47)		Time Effects^c^	Group by Time Effects^c^
	Baseline, mean (SD)^d^	Follow-up, mean (SD)^d^	Baseline, mean (SD)^d^	Follow-up, mean (SD)^d^	*P* Value	*P* Value
**Anthropometrics and blood pressure**						
Body mass index, kg/m^2^	35.9 (7.2)	34.7 (7.2)	36.6 (8.1)	36.4 (8.0)	<.001	<001
Weight, kg	99.6 (24.1)	96.5 (23.9)	98.0 (20.4)	97.6 (20.5)	<.001	<001
Waist circumference, cm	109.1 (15.7)	106.2 (15.9)	110.7 (16.5)	110.0 (17.1)	<.001	.01
Systolic blood pressure, mm Hg	132.6 (20.6)	131.2 (21.0)	128.2 (14.7)	126.4 (16.0)	.15	.84
Diastolic blood pressure, mm Hg	80.2 (12.1)	80.4 (12.7)	78.1 (8.0)	77.1 (8.0)	.55	.40
**Leisure-time physical activity (min/wk)**						
Moderate activity	37.3 (67.6)	74.1 (114.6)	21.8 (57.9)	49.7 (80.5)	.003	.67
Vigorous activity	36.5 (89.1)	49.2 (96.6)	9.7 (29.7)	25.8 (52.3)	.06	.82
Strength activity	8.9 (25.9)	14.6 (29.7)	11.5 (38.6)	7.6 (19.5)	.79	.17
**Dietary intake**						
Sugar, teaspoon	17.3 (8.9)	13.5 (7.5)	17.9 (10.2)	15.6 (9.6)	<.001	.24
Calcium, mg	671.7 (111.2)	670.0 (110.2)	675.9 (86.8)	671.4 (91.2)	.70	.87
Fiber, g	20.8 (5.7)	20.7 (5.4)	21.6 (4.9)	21.3 (5.1)	.75	.80
Fruits and vegetables, servings	4.5 (2.3)	4.6 (1.8)	4.5 (2.2)	4.8 (2.2)	.35	.53
Fruits and vegetables, cup	5.4 (8.2)	5.0 (4.1)	6.0 (9.5)	6.8 (9.0)	.75	.46
Dairy, servings	1.0 (0.5)	1.1 (0.9)	1.1 (0.7)	1.1 (0.7)	.28	.31
**Psychosocial measures for physical activity**						
Self-efficacy^e^	75.07 (13.88)	71.23 (15.24)	73.51 (17.43)	70.01 (19.46)	.003	.89
Family support^f^	2.96 (1.22)	2.98 (1.26)	2.81 (0.99)	2.86 (1.00)	.57	.83
Friend support^f^	3.42 (0.85)	3.48 (0.90)	3.11 (1.09)	3.14 (1.01)	.46	.76
**Psychosocial measures for nutrition**						
Self-efficacy^e^	81.95 (12.53)	80.70 (15.26)	81.78 (14.07)	80.21 (16.00)	.19	.88
Family support^f^	2.61 (0.85)	2.61 (0.85)	2.53 (0.74)	2.69 (0.84)	.15	.15
Friend support^f^	2.96 (0.89)	3.08 (0.99)	2.77 (0.82)	2.89 (0.89)	.05	>.99

a Twice-weekly access to group fitness classes plus weekly nutrition and physical activity education sessions.

b Twice-weekly access to group fitness classes.

c Calculated by *F*-test for analysis of variance.

d Groups 1 and 2 were not significantly different (*P * < .05) at baseline.

e 100-point continuum scale (0 = certain I cannot, 100 = certain that I can). Defined as confidence in being physical active and eating healthfully under different conditions.

f Five-point Likert scale (1 = strongly disagree, 5 = strongly agree). Defined as the social influence of people on physical activity and eating behaviors.

**Figure 2:**
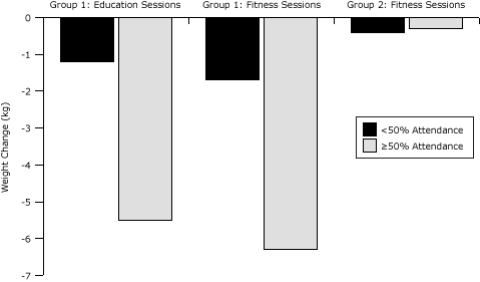
Weight change by attendance at group education sessions and group fitness sessions (N = 91), Better Together Healthy Caswell County, North Carolina, 2011.

**Figure 3:**
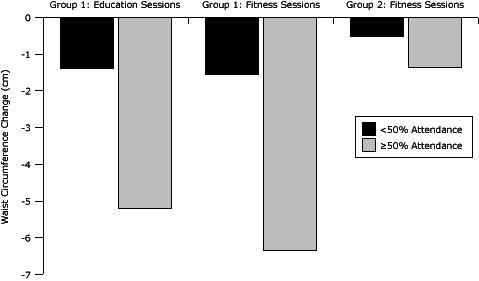
Change in waist circumference by attendance at group education sessions and group fitness sessions (N = 91), Better Together Healthy Caswell County, North Carolina, 2011.

Group 1 had significant weight loss effects by level of attendance at sessions (<50% or ≥50%): education attendance (*F* = 14.7, *P* < .001) and fitness attendance (*F* = 18.5, *P* < .001) (Figure 2). Significant effects for waist circumference were found by education attendance (*F* = 11.6, *P *< .001) and fitness attendance (*F* = 5.8, *P* = .02) (Figure 3). For group 2, effects were not significant by group fitness attendance (Figure 3).

Waist circumference differed significantly by percentage of education and fitness sessions attended. Group 1 members who attended 50% or more of the education sessions decreased waist circumference more than did those who attended less than 50% of the sessions, *P* < .001. Group 1 members who attended 50% or more of the fitness sessions also had greater changes in waist circumference than did those who attended less than 50% of the sessions, *P* = .02. Fifty-eight participants completed the qualitative exit questionnaire. Responses indicated that group fitness was well received and Zumba was the favored activity. The nutrition classes were also well received, although many participants requested a personalized planning component. Group cohesion and accountability also contributed to an enjoyable experience. Participants recommended that future programs have more sessions and serve more people.

## Discussion

Our findings are congruent with those of systematic reviews that found that exercise plus diet programs tend to be superior at producing and maintaining weight loss compared with programs that promote weight loss through diet alone or physical activity alone (25,26). Although both groups reported increases in moderate physical activity and experienced significant changes in weight, participants in the intervention group (group 1), who could attend weekly classes on healthy eating and physical activity in conjunction with twice-weekly group fitness classes, had significantly greater weight loss than did participants who had access only to fitness classes (group 2). On average, participants in group 1 lost about 3% of their baseline weight, whereas those in group 2 lost <0.5%. Clinically meaningful weight loss is typically defined as a 5% to 10% reduction in baseline weight, an amount that improves numerous risk factors associated with obesity (26,27). Although our study did not reach this threshold of clinical significance, our 15-week pilot trial was shorter than most weight loss programs (eg, 6–18 months); longer programs generally result in more weight loss (26,27). Likewise, although on average participants did not achieve CDC’s recommendation of 150 min/wk of moderate-to-vigorous physical activity, participants’ combined amount of moderate and vigorous physical activity nearly doubled. These improvements resulted in a naturally occurring community setting, where conditions were much less controlled than in clinical trials.

Although our primary outcomes improved as hypothesized, several secondary outcomes did not change. For example, we hypothesized a greater improvement in dietary variables among the participants randomized to receive the ESMMWL curriculum (group 1). Null findings may be due, in part, to limitations of the screener used to assess dietary changes (19). The screening instrument was selected because of its low respondent burden and ease of use and scoring; however, the instrument was developed for use at the population level and was not validated for use at the individual level. Future studies should include dietary methods sensitive enough to detect changes at the individual level. Similarly, we hypothesized that participants receiving the ESMMWL curriculum would achieve greater improvements in the psychosocial variables related to healthy eating. Although this curriculum was grounded in the theory of planned behavior, related measures to evaluate changes in theoretical constructs associated with the curriculum have not been developed (15–17). The chosen instruments and underlying constructs (self-efficacy and family and friend support) were determined to be the most culturally relevant for the participants and consistent with the efforts of the DRPHC physical activity subcommittee. Future research is needed to develop and evaluate culturally appropriate psychosocial instruments that are matched to the goals and underlying theoretical constructs of the ESMMWL curriculum. In addition, self-efficacy for physical activity significantly decreased from baseline to follow-up among both groups. This phenomenon has been observed in other behavioral trials, as participants engage in physical activity and realize the difficulty in maintaining such efforts (28).

The relationship among levels of participation and outcomes also help inform future sustainable programs. The attendance expectations for the 15-week program were high. Although arguably necessary to help meet physical activity recommendations and provide adequate education time, engaging participants in structured activities 3 nights a week may be unrealistic. Nonetheless, group 1 participants who attended more than 50% of the sessions achieved clinically significant weight reductions (5.5%–6.3%) compared with those who attended less than 50% of the sessions. This positive relationship between high attendance and the desired outcome is consistent with findings of other studies (29). Future study is needed to develop scalable approaches that can provide educational content and motivational support yet adequately reach geographically dispersed residents with hectic lifestyles.

In the qualitative exit questionnaire, several participants spoke of the need for personal customization of both the fitness and eating regimens. Many participants who were motivated to be physically active but could not attend the fitness sessions regularly because of scheduling conflicts expressed frustration over not receiving recognition for physical activity performed outside of the group fitness sessions. To address these concerns, regular collection and review of the participants’ records and diaries of all food and physical activity behaviors, along with a feedback loop and reward system, should be considered for future programs. Furthermore, many members of group 1 commented on the relationships they built with their peers. Incorporating evidence-based principles on group dynamics (30) may strengthen the delivery process, enhance the participants’ experiences and retention rates, and improve future intervention effects.

In the context of guiding CBPR principles, the less tangible outcomes are perhaps the most important (8,9). For most DRPHC stakeholders and participants involved in this study, this was their first exposure to any aspect of research. The collaborative process involved in this study helped create an atmosphere of shared ownership in the research process and in the evaluation components. Study procedures, enrollment and participation rates, and outcome data were disseminated at the monthly DRPHC coalition meetings. This procedure allowed the researchers to understand the unique needs and dynamics of the community and make protocol adjustments accordingly, and it helped community stakeholders gain an appreciation for the research process. Collaborating with and gaining trust of vulnerable communities are essential elements of CBPR and are necessary to promote program sustainability. Obesity prevalence among the enrolled community sample and the lack of regional physical activity resources (14) signify the need for evidence-based weight-management programs in this region. The relationships forged through this pilot study created a critical alliance that was used as leverage when proposals were submitted for other obesity-related intervention grants.

This study has limitations. These findings may be generalizable only to women and individuals who are motivated to change behavior and lose weight. Our attrition rates also have the potential to bias our results. We explored this concern by using both intent-to-treat and present-at-follow-up analyses, and findings did not vary by analytical approach. Nevertheless, we purposefully chose to present data from the intent-to-treat analysis and to account for all enrolled participants because this method is the more conservative and produces the more generalizable estimate of effects. Furthermore, although the study was adequately powered to determine primary outcome effects, it may be underpowered to determine secondary outcome effects. Likewise, although we used validated instruments, they may not have been the best fit for the curriculum. Despite these limitations, this pilot study sufficiently achieved the desired outcomes of informing the feasibility of implementing larger scale community-based experimental interventions in the region and promoting collaboration and resource-sharing among members of the CBPR coalition. Future programs of similar design should attempt to account for potential contamination across groups (eg, group randomization, postassessments to determine information sharing with acquaintances in different groups).

Our pilot findings suggest that giving people access to 15 weeks of free group fitness can increase minutes of moderate physical activity in a region that has health disparities and lacks physical activity resources, but such access alone is insufficient to improve weight outcomes. To effectively improve weight outcomes, access to both physical activity and educational programs is needed. This study also signifies a successful collaboration among several community–academic partners engaged in a CBPR coalition. Future studies are needed to determine the long-term clinical effectiveness and cost-effectiveness of similar efforts and the ability of the DRPHC coalition and local organizations to sustain programs that provide access to free physical activity and weight management.
